# Characterization of m6A Methylation Modification Patterns in Colorectal Cancer Determines Prognosis and Tumor Microenvironment Infiltration

**DOI:** 10.1155/2022/8766735

**Published:** 2022-06-01

**Authors:** Qingfang Yue, Yuan Zhang, Fei Wang, Fei Cao, Jun Bai, Xianglong Duan

**Affiliations:** ^1^Department of Medical Oncology, Shaanxi Provincial People's Hospital, Xi'an, 710068 Shaanxi, China; ^2^Institute of Medical Research, Northwestern Polytechnic University, Xi'an, 710072 Shaanxi, China; ^3^Department of Gynecology, Shaanxi Provincial People's Hospital, Xi'an, 710068 Shaanxi, China; ^4^Second Department of General Surgery, Shaanxi Provincial People's Hospital, Xi'an, 710068 Shaanxi, China; ^5^Second Department of General Surgery, Third Affiliated Hospital of Xi'an Jiaotong University, Xi'an, 710068 Shaanxi, China

## Abstract

Cumulative studies have suggested that dysregulation of m6A regulators and immunity is highly linked to the prognosis of patients with cancer. However, the potential contribution of m6A modification patterns to the tumor microenvironment (TME) and the therapeutic efficacy of immunotherapy for colorectal cancer (CRC) remain elusive. A comprehensive analysis of the m6A modification profiles of 458 patients with CRC was performed by clustering 21 genes encoding m6A methylation regulators and linking the m6A modification pattern with TME characteristics. Using principal component analysis (PCA), a risk model was constructed to quantify individual m6A modification patterns in patients with CRC. The results indicated that the expression profiles and genetic mutations of 21 genes encoding m6A methylation regulators in CRC were characterized by a high degree of heterogeneity. Three m6A clusters had significant differences in prognosis, m6A modification patterns, and TME characteristics. Furthermore, a risk model, termed m6Ascore, was developed by PCA to quality m6A methylation patterns at an individual level. The m6Ascore could stratify patients into high- and low-m6Ascore groups. Further analyses demonstrated that the m6Ascore had a good predictive performance for overall survival and clinical efficacy of immunotherapy in patients with CRC. Finally, the predictive value of the model was validated by external cohorts. In conclusion, the comprehensive characterization of m6A methylation modification patterns might contribute to our understanding of the TME in CRC and the development of personalized antitumor immunotherapy in the future.

## 1. Introduction

Colorectal carcinoma (CRC) is the third most commonly diagnosed cancer and is the second leading cause of cancer-related deaths globally [1]. Although current treatments such as targeted therapy, immunotherapy, and precision treatment have been applied for the treatment of CRC [2, 3], the clinical outcomes are unsatisfactory, and the prognosis of patients with CRC remains bleak. The prognosis of patients with CRC varies widely even among patients with the same stage and therapeutic regimen, largely attributed to the highly heterogeneous nature of CRC [4]. In the current era, immunotherapy using immune checkpoint inhibitors (ICIs) is an encouraging strategy for the treatment of solid tumors, including CRC [5–10]. The heterogeneity of CRC at the genetic and molecular levels has significant ramifications in terms of immunotherapy outcomes [11]. In CRC, microsatellite instability [12], tumor mutational burden (TMB) [13], and DNA polymerase epsilon mutations [14] have emerged as major markers to predict the responses to immunotherapy. However, these indicators do not provide accurate predictions for the current ICIs, thereby facilitating the development of more accurate and reliable biomarkers.

As a common chemical modification of eukaryotic messenger ribonucleic acids (mRNAs), N6-methyladenosine (m6A) can affect various essential biological processes by regulating the expression of target genes [15, 16]. m6A-regulated proteins consist of “writers” (WTAP, METTL3, and METTL14), “erasers” (ALKBH5 and FTO), and “readers” (IGF2BPs and YTHDFs) [17–19]. Currently, numerous studies have offered evidence that m6A regulators perform crucial functions in modulating the maturation, translation, and degradation of RNAs (including mRNAs and noncoding RNAs). Cumulative studies have revealed that m6A regulator dysregulation is correlated with apoptosis, proliferation, self-renewal, developmental defects, malignant tumor progression, and tumor microenvironment (TME) [20–22]. TME refers to the environment surrounding tumor cells, including perivascular cells, immune cells, fibroblasts, molecules, extracellular matrix, and additional stromal components. Currently, in CRC, several reports have suggested that m6A methylation regulators play a crucial role in the tumor immune microenvironment (TIME), which is defined as the immune and the immune-related constituents of the TME. For instance, Cai et al. highlighted that downregulation of the m6A “writer” METTL14 represents an unfavorable patient prognosis and that its low expression level may result in the downregulation of m6A modifications and reduction of the level of immune cell infiltration [23]. Approximately 85% of patients with CRC exhibit mismatch-repair-proficient or microsatellite instability-low (pMMR-MSI-L) tumors, and such patients are poorly responsive to immunotherapy. Wang et al. reported that depletion of the methylation transferases METTL3 and METTL14 could suppress m6A modification and enhance responses of pMMR-MSI-L CRC to anti-PD-1 therapy. Furthermore, *in vivo* experiments suggested that METTL3- or METTL14-deficient tumors were associated with increased abundance of cytotoxic tumor-infiltrating CD8^+^ T cells and significantly enhanced the secretion of IFN-*γ*, CXCL9, and CXCL10 in TME [24]. According to Tsuruta et al., the m6A “erasers” gene-FTO could regulate PD-L1 expression in colon cancer cells in an IFN-*γ* signaling-independent manner [25]. However, to date, most studies have only focused on the role of single m6A regulators in the regulation of the TIME and have failed to provide a comprehensive perspective on how m6A modification patterns contribute to the TIME.

In this study, we comprehensively investigated m6A modification characteristics and identified three m6A modification patterns (m6Aclusters) with distinct survival benefits, TME immune cell infiltrations, and transcriptome characteristics. Moreover, we constructed an m6A regulator-associated gene signature, m6Ascore, which serves as a risk model for assessing individual m6A modification patterns. Therefore, the m6Ascore is a promising biomarker for predicting prognosis and clinical response to ICIs in patients with CRC.

## 2. Materials and Methods

### 2.1. Date Collection

The whole workflow of our present study is displayed in Supplementary Figure S1. RNA sequencing (RNA-seq) data for gene expression analysis, genetic mutations, and clinical information of patients with colon carcinoma (*n* = 331) and rectal carcinoma (n =127) were downloaded from The Cancer Genome Atlas (TCGA, http://cancergenome.nih.gov/). Gene expression data and its corresponding clinical information were obtained from the GSE17536 (*n* = 177) and GSE78220 (*n* = 27) datasets in the Gene Expression Omnibus (GEO) database. The Broad GDAC Firehose (http://gdac.broadinstitute.org/) was retrieved to obtain information on copy number variations (CNVs). In addition, the IMvigor210 cohort, a dataset of advanced urothelial cancer treated with PD-L1 inhibitor (atezolizumab), was collected using the “IMvigor210” R package [26]. GSE78220 (*n* = 27), a dataset of metastatic melanoma treated with anti-PD-1 antibody (pembrolizumab), was collected from the GEO database [27]. The above two immunotherapeutic datasets were collected to assess our findings.

### 2.2. Analysis of Somatic Mutations

Gene somatic mutation data (MAF files) containing the mutation profile of the CRC cohort were obtained from TCGA database. The visualization procedure was performed with the aid of the “maftools” R package [28].

### 2.3. Correlation Analysis between m6A Regulators and Immune Infiltrations

The “CIBERSORT” package in R was employed to assess the infiltration levels of 22 distinct immune cell types across m6A regulators and m6A clusters [29].

### 2.4. Unsupervised Clustering for m6A Regulators

A total of 21 m6A modulators were obtained from previous studies [30] (Supplementary Table S1) and analyzed to identify the characteristics of m6A modification. Unsupervised clustering analysis was performed using the “ConsensusClusterPlus” R package [31], and tumors were sampled for 1000 bootstrap replications to ensure stability. The cluster number was calculated by the area under the cumulative distribution of the function curve, and the *k*-means method was used with the Euclidean distance metric. Distinct m6A modification profiles were identified according to the expression values of 21 m6A regulators, and patients were classified for subsequent analysis.

### 2.5. Identification of Differentially Expressed Genes Corresponding to m6A Clusters

The “limma” R package was applied to identify differentially expressed genes (DEGs) across various m6A clusters [32]. DEGs were considered significantly different between groups in the case where ∣ log2 (fold change)  | >1 and adjusted *P*value < 0.05.

### 2.6. Gene Set Enrichment Analysis and Gene Enrichment Functional Annotation

Gene set enrichment analysis (GSEA), an approach for interpreting gene expression data, was conducted to thoroughly comprehend the distinct biological functions underlying m6A modification patterns [33]. The Molecular Signature Database (MSigDB) for GSEA (https://www.gsea, http://msigdb.org/gsea/index.jsp) was used to acquire the gene set “h.all.v7.2.symbols.gmt.” The enrichment as well as modeled enrichment scores were calculated using the “GSEA” R package. Values with adjusted *P* < 0.05 were considered statistically significant. The clusterProfiler package in R was used to conduct Gene Ontology (GO) analysis of m6A-associated DEGs. Values with *P* < 0.05 were considered statistically significant.

### 2.7. Correlation Analysis between m6A Clusters and Immunomodulators

A total of 75 immunomodulators (IMs) were extracted from previous studies [34]. One-way analysis of variance (ANOVA) and Kruskal–Wallis tests were used to investigate the differences in IM potency among different clusters.

### 2.8. Dimension Reduction and Construction of the m6Ascore Model

The m6Ascore model was constructed based on m6A cluster-associated DEGs. First, a total of 194 m6A cluster-associated DEGs were selected for subsequent analysis. Unsupervised clustering was performed to define DEG clusters. Next, principal component analysis (PCA) was performed for dimension reduction, and the final weight values of PC1 and PC2 were used to construct the m6Ascore model with the formula as follows [35]: m6Ascore = ∑(PC1*i* + PC2*i*), where “*i*” represents the expression of m6A regulator cluster-associated DEGs.

### 2.9. Statistical Analysis

The correlation coefficient was evaluated by Spearman's correlation analysis. The optimal cutoff value for each cohort was determined by the “survminer” R package. Statistical significance of comparisons between two groups was computed by Student's *t*-test or the Wilcoxon signed-rank test. The Kruskal-Wallis test and one-way ANOVA were used for the purpose of examining statistical differences among the three groups. The Kaplan-Meier method was employed to perform prognostic analysis, whereas the log-rank test was used to assess if there were any differences among the groups. A protein-protein m6A regulator interaction (PPI) network was constructed using the STRING database [36]. All statistical *P* values were double-sided. Statistical significance was set at *P* < 0.05. All statistical analyses were performed using R 3.6.1 (https://www.r-project.org/).

## 3. Results

### 3.1. Characterization and Clinical Value of m6A Regulators in CRC

**Figure 1:**
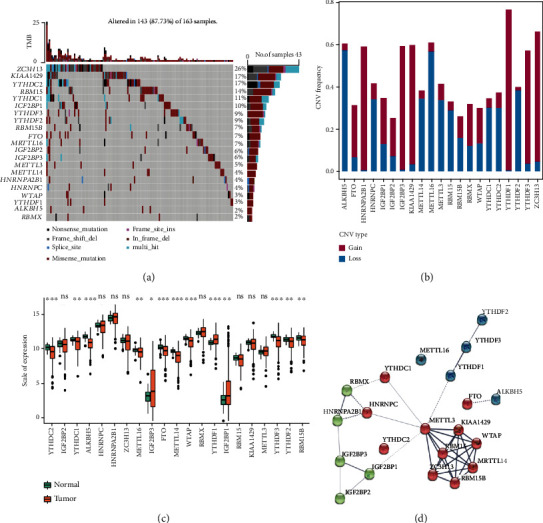
Multiomics characterization of 21 m6A methylation regulators in The Cancer Genome Atlas-colorectal cancer (TCGA-CRC) dataset. (a) A waterfall plot of 21 m6A methylation gene mutations in TCGA-CRC cohort. (b) The frequency of copy number variations (CNVs) in genes encoding m6A methylation regulators. (c) A boxplot showing the differences in gene expression of 21 m6A methylation regulators in tumor versus normal tissues (orange denotes the tumor sample, and green denotes the normal sample). ^∗∗∗^*P* < 0.001, ^∗∗^*P* < 0.01, and ^∗^*P* < 0.05. (d) A network plot of protein-protein interactions among 21 m6A methylation regulators.

In the present study, 21 m6A-related genes including “writers” (*ZC3H1*3, *VIRMA*, *WTAP*, *RBM15*, *RBM15B*, *METTL3*, *METTL14*, and *METTL16*), “erasers” (*ALKBH5* and *FTO*), and “readers” (*IGF2BP1*, *IGF2BP2*, *IGF2BP3*, *RBMX*, *HNRNPC*, *HNRNPA2B1*, *YTHDC1*, *YTHDC2*, *YTHDF1*, *YTHDF2*, and *YTHDF3*) were identified from previous studies [30]. In TCGA-CRC dataset, the overall mutation frequency of the above 21 RNA methylation-related genes was high, with 143 of 163 (87.73%) samples having mutations in 21 genes encoding m6A regulators. The genes undergoing the highest mutation frequencies were *ZC3H13* (26%), *KIAA1429* (17%), and *YTHDC2* (17%) (Figure 1(a)). Then, we investigated the CNV of 21 genes encoding m6A regulators and found that CNVs were frequent in TCGA-CRC dataset. The CNV of *ALKBH5*, *HNRNPA2B1*, *IGF2BP3*, *KIAA1429*, *METTL16*, *YTHDF1*, *YTHDF3*, and *ZC3H13* was more than 50%. Among them, *ALKBH5* and *METTL16* primarily exhibited deletions, and the other genes mostly exhibited amplification (Figure 1(b)). At the transcriptomic level, we compared the differential expression of genes encoding 21 m6A regulators in tumor and healthy samples, and the results showed that 13 out of 21 genes were significantly differentially expressed. The genes *IGF2BP1*, *IGF2BP3*, and *YTHDF1* were significantly upregulated in tumor samples, whereas the genes *YTHDC1*, *YTHDC2*, *ALKBH5*, *METTL14*, *METTL16*, *FTO*, *WTAP*, *YTHDF2*, *YTHDF3*, and *RBM15B* were significantly downregulated in tumor tissues (Figure 1(c)). Furthermore, the PPI network constructed using STRING implies extensive protein interactions among these regulators. The interactions and prognostic relevance of the 21 m6A regulators in patients with CRC are illustrated by the regulatory network shown in Figure 1(d), and the results suggested a positive coexpression profile in most genes. Taken together, the above results indicate that the genetic and expression differences of m6A regulators are highly heterogeneous in CRC, suggesting that differences in the expression of m6A regulators might have a key role in the onset and progression of CRC.

### 3.2. Prognosis and Immune Landscape of m6A Regulators

**Figure 2:**
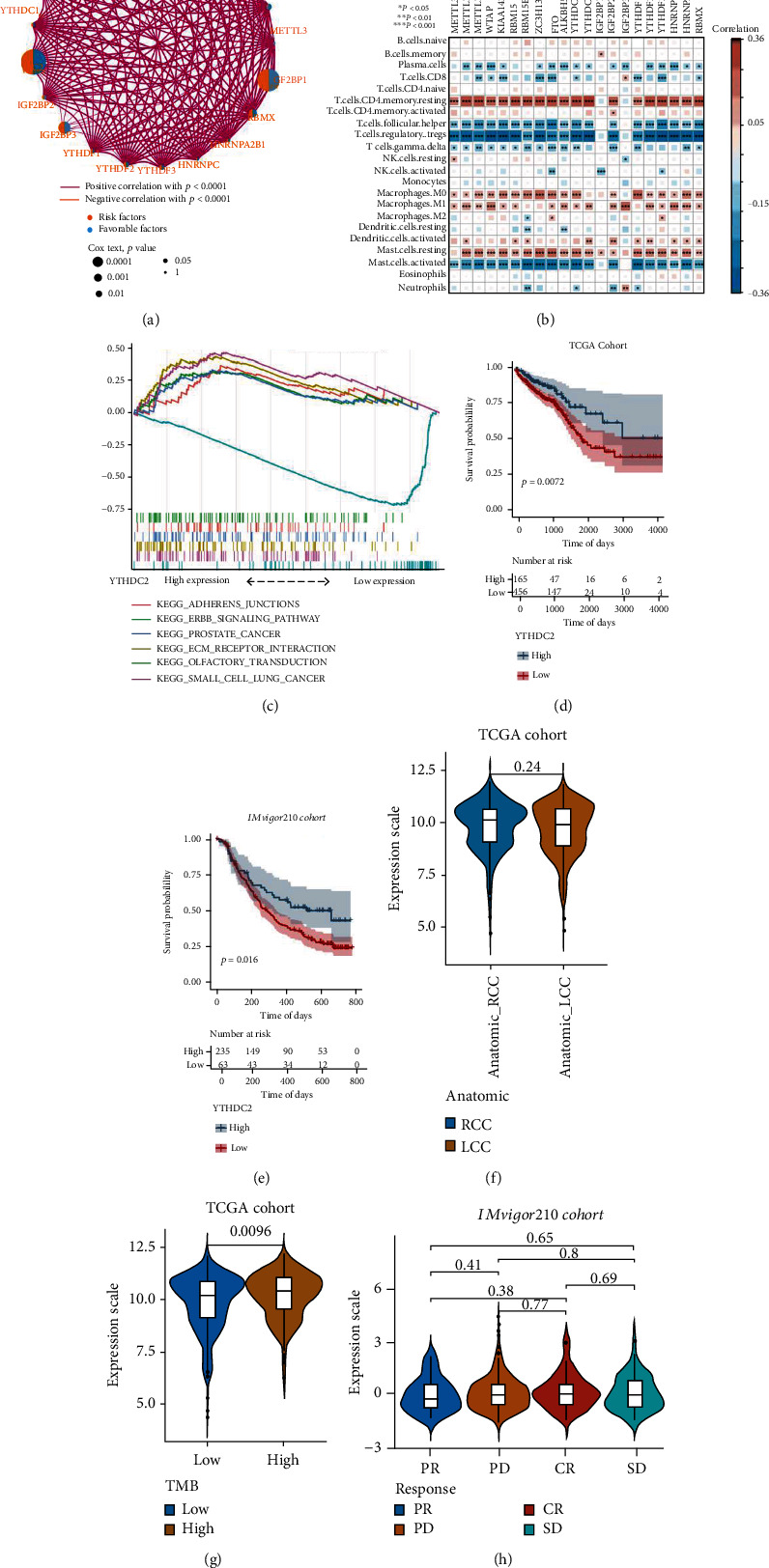
Relationship between m6A regulator expression and tumor immune cell infiltration in The Cancer Genome Atlas-colorectal cancer (TCGA-CRC) dataset. (a) Correlation and prognosis network of 21 m6A regulators in patients with CRC. (b) Correlation heat map between immune cell infiltrates and 21 m6A regulator expression. (c) Gene set enrichment analysis (GSEA) for high and low YTHDC2 expression status. (d) Correlation between overall survival (OS) and YTHDC2 expression in TCGA-CRC cohort (log-rank test, *P* = 0.0072). (e) Correlation between OS and YTHDC2 expression in IMvigor210 cohort (log-rank test, *P* = 0.016). (f) Differences in YTHDC2 expression across anatomical locations in TCGA-CRC dataset. (g) Differences in YTHDC2 expression and tumor mutational burden in TCGA-CRC dataset. (h) A boxplot showing relative YTHDC2 expression in different clinical response groups.

First, the coexpression and prognostic significance of the 21 m6A regulators in TCGA-CRC cohort were analyzed and further visualized in the network (Figure 2(a)). The results revealed that most of the m6A regulators were significantly positively correlated.

Recent evidence has demonstrated that m6A modifications are involved in tumor immunity [22]. For the purpose of further analyzing the association between m6A modifications and the TME, we investigated the association of 21 m6A gene expression profiles with the infiltration of 22 distinct immune cell types. An extensive correlation was found between the expression of m6A regulators and the infiltration of most immune cell subtypes, with significant differences between the expression of different genes and immune cell infiltration (Figure 2(b)). The m6A eraser *YTHDC2* was determined as a tumor suppressor in the above analysis. Results of GSEA demonstrated a predominant enrichment of ADHERENS JUNCTION, ECM RECEPTOR INTERACTION, ERBB SIGNALING PATHWAY, PROSTATE CANCER, and SMALL CELL LUNG CANCER in the *YTHDC2* high-expression group and a predominant enrichment of OLFACTORY TRANSDUCTION in the *YTHDC2* low-expression group (Figure 2(c)). Results of survival analysis indicated a significantly longer overall survival (OS) duration in patients belonging to the *YTHDC2* high expression group compared to those belonging to the *YTHDC2* low expression group in both TCGA-CRC and IMvigor210 cohorts (Figures 2(d) and 2(e)). The anatomical location of the primary tumor is also recognized as an important factor for colon cancer prognosis [37]. As illustrated in Figure 2(f), no significant differences were recorded for the expression of the *YTHDC2* gene in primary tumors with different anatomical locations. TMB is an important indicator to assess the efficacy of immunotherapy for CRC [13]. The expression of the *YTHDC2* gene was significantly higher in the high-TMB group than in the low-TMB group (Figure 2(g)). Given the crucial role played by *YTHDC2* in tumor immunity, we thoroughly investigated if *YTHDC2* expression could predict immunotherapeutic responses. Unfortunately, in the IMvigor210 dataset, no significant difference was discovered in terms of the expression of *YTHDC2* in patients with therapeutic response compared to patients without therapeutic response (Figure 2(h)).

Taken together, these results disclose a significant correlation between the expression of m6A methylation regulators and the TME. The expression of the m6A eraser YTHDC2 is closely associated with immune cell infiltration and might be a favorable biomarker for prognosis. However, the prediction significance of YTHDC2 in terms of immunotherapeutic efficacy still needs further validation.

### 3.3. Identification of m6A Modification Characteristics Based on 21 m6A Regulators

**Figure 3:**
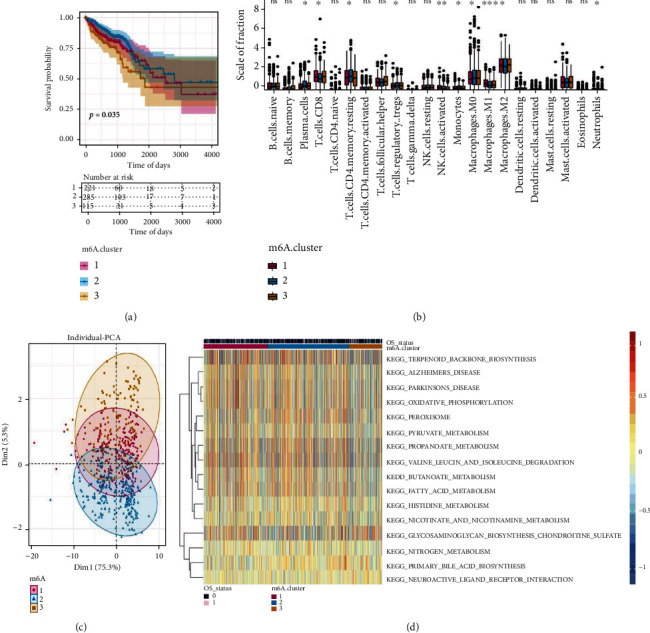
Identification and functional enrichment analysis of m6A clusters in The Cancer Genome Atlas-colorectal cancer (TCGA-CRC) dataset. (a) Overall survival curves among three m6A clusters. (b) A scatter plot of principal component analysis (PCA) of 21 m6A methylation regulators. (c) Boxplot depicting the relative immune cell infiltrations among three m6A clusters. (d) Kyoto Encyclopedia of Genes and Genomes (KEGG) analysis of gene sets among three m6A clusters.

m6A regulators might be responsible for the CRC heterogeneity, and they are tightly correlated with TME. To further identify novel m6A regulator profiles, we performed unsupervised clustering based on the expression of 21 m6A regulators in TCGA-CRC data. As demonstrated in Supplementary Figure S2, three clusters showed the best clustering performance, and patients were stratified into m6Acluster-1 (*n* = 221), cluster-2 (*n* = 285), and cluster-3 (*n* = 115). Survival analysis revealed that the three m6A clusters had significant differences in the OS. The OS duration of patients in m6Acluster-1 and m6Acluster-2 was significantly longer than that of patients in m6Acluster-3 (Figure 3(a)). The PCA algorithm was employed to visualize the expression profile of 21 m6A regulators, and the results indicated that three clusters could be well distinguished (Figure 3(b)). Therefore, the classification of the m6A clusters was reasonable.

Subsequently, we investigated the differences in immune cell infiltrations among m6Aclusters and found that the infiltration levels of CD8 T cells, activated NK cells, M1 macrophages, M2 macrophages, and neutrophils were significantly higher in m6Acluster-1 and m6Acluster-3 than in the m6A-2 cluster. However, the infiltration levels of plasma cells, M0 macrophages, resting CD4-positive memory T cells, regulatory T cells, and monocytes were higher in m6Acluster-2 than in m6Acluster-1 and m6Acluster-3 (Figure 3(c)). Next, we examined the differences in the role of signaling pathways across the three clusters, and the results indicated that m6Acluster-1 and m6Acluster-3 were mainly enriched in KEGG TERPENOID BACKBONE BIOSYNTHESIS, KEGG ALZHEIMERS DISEASE, KEGG PARKINSONS DISEASE, KEGG OXIDATIVE PHOSPHORYLATION, and KEGG PEROXISOME and that m6Acluster-2 was mainly enriched in KEGG NITROGEN METABOLISM, KEGG PRIMARY BILE ACID BIOSYNTHESIS, and KEGG NEUROACTIVE LIGAND RECEPTOR INTERACTION (Figure 3(d)).

### 3.4. Clinical and Transcriptomic Characteristics of the Three m6A Clusters

**Figure 4:**
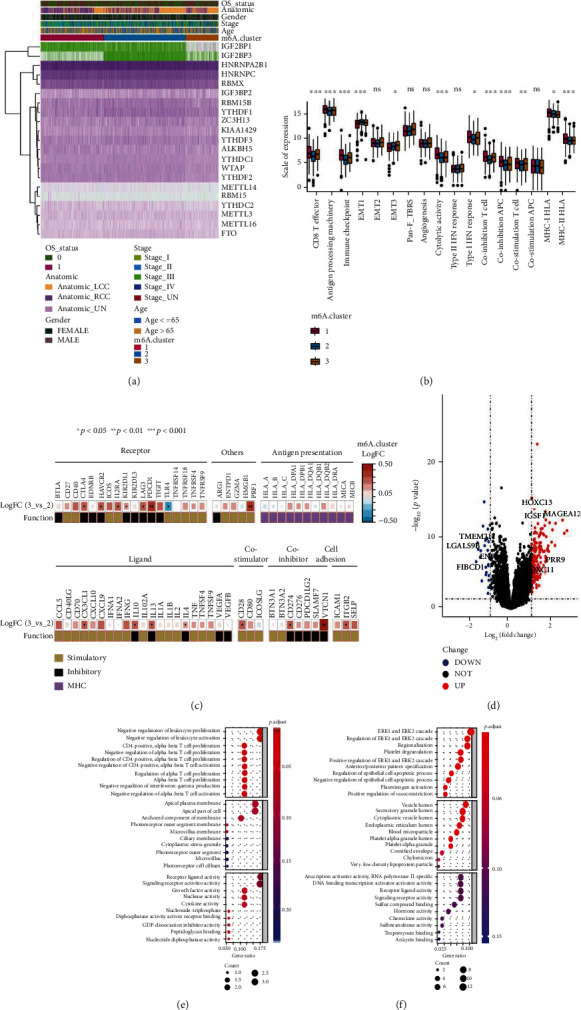
Differential expression of immune signatures among three m6A clusters. (a) Heat map depicting the expression profile of 21 m6A regulators. (b) A boxplot of relative immune signatures among three m6A clusters. (c) Differential expression of immunomodulatory molecule expression among tumor m6A clusters. (d) A volcano plot of differential expression gene analysis among three m6A clusters. (e) Gene Ontology (GO) enrichment analysis of upregulated gene sets among m6A clusters. (f) GO enrichment analysis of downregulated gene sets among m6A clusters.

First, we explored the role of m6A regulators in the classification of m6A clusters. The heat map of m6A-associated genes showed that *IGF2BP1* and *IGF2BP3* played a major role in the classification process (Figure 4(a)). Immune infiltration-associated signatures have a significant role in TME. Therefore, it is necessary to thoroughly probe into the correlation between m6A clusters and various immune infiltration-associated signatures as established by Mariathasan et al. [26]. We first investigated differences in the expression of these immune infiltration-associated signatures in m6A clusters, and the results indicated that m6Acluster-1 and m6Acluster-3 were enriched in immune activation signatures such as CD8 T effector, antigen processing machinery, immune checkpoint, cytolytic activity, type I IFN response, coinhibition T cell, coinhibitory APC, costimulation T cell, MHC-I HLA, and MHC-II HLA. Moreover, m6Acluster-3 was enriched in stroma activation phenotype signatures, including EMT1, EMT3, and pan-F-TBRS (Figure 4(b)). Furthermore, we performed differential expression analysis of IMs among the three clusters and found that multiple genes showed significant differential expression (Figure 4(c)). A comprehensive analysis of the above results and the characteristics of immune cell infiltrations and patient prognosis in the three subtypes revealed that m6Acluster-1 is an immune-inflamed phenotype associated with elevated infiltration levels of immune cells and that m6Acluster-3 is an immune-excluded phenotype associated with the infiltration levels of multiple immune cell types. However, these cells are reserved in the surrounding nests of tumor cells instead of penetrating their stroma. Furthermore, m6Acluster-2 was an immune-desert phenotype characterized by a few immune cell infiltrates and a suppressive immunological landscape [38].

To further uncover the potential biological profile of distinct m6A patterns, DEGs among m6A clusters were analyzed. A total of 194 genes were identified, out of which 167 genes were significantly upregulated and 27 were significantly downregulated (Figure 4(d)). GO analysis was performed on the upregulated and downregulated DEGs, and the top 10 pathways enriched in the three functional categories (biological process (BP), cellular component (CC), and molecular function (MF)) are shown in bubble plots. As shown in Figures 4(e) and 4(f), the majority of the enriched pathways are linked to BPs such as differentiation of immune cells, ligand and receptor activity of growth factors and cell membranes, and stromal-related and vesicle formation. The above results indicate the different clinical and transcriptomic features of the m6A clusters.

### 3.5. Construction of the m6Ascore Model

**Figure 5:**
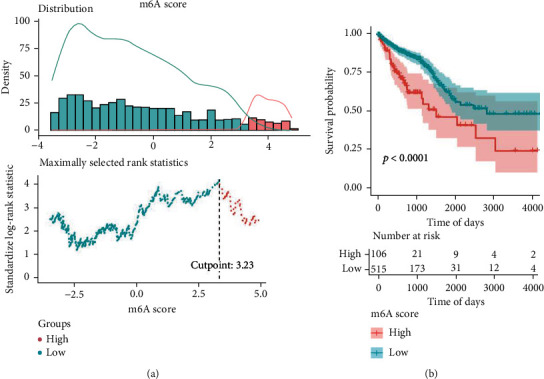
The optimal gradient for the m6Ascore (m6Ascore) among different clinical groups. (a) Correlation between density statistics of m6Ascore distribution and the determination of optimal threshold. (b) Survival curves between high- and low-m6Ascore groups.

Unsupervised clustering analysis was performed using the obtained expression profiles of 194 tumor m6A cluster-associated DEGs. As a result, the tumor samples of TCGA-CRC could be categorized into three m6Acluster-associated differentially expressed gene clusters (DEG-cluster), named Gene cluster-1, Gene cluster-2, and Gene cluster-3. Results of the prognostic analysis indicated significant survival differences among DEG clusters (Supplementary Figure S3). The above analysis was performed based on m6A methylation modifications, reflecting accurately the expression profiles of m6A regulators in CRC. Furthermore, based on the DEGs among m6A clusters, we could construct a risk score system by PCA algorithm, referred to as the m6Ascore, for the purpose of quantifying m6A modification profiles in individual patients with CRC. The optimal density threshold of the m6Ascore associated with survival was calculated using the “survminer” R package, with a threshold value of 3.23. This threshold value was used to classify TCGA-CRC samples into high- and low-m6Ascore groups (Figure 5(a)). The findings from the Kaplan-Meier survival analysis demonstrated an improved prognosis condition among patients in the low-m6Ascore group compared to those in the high-m6Ascore group (Figure 5(b)).

**Figure 6:**
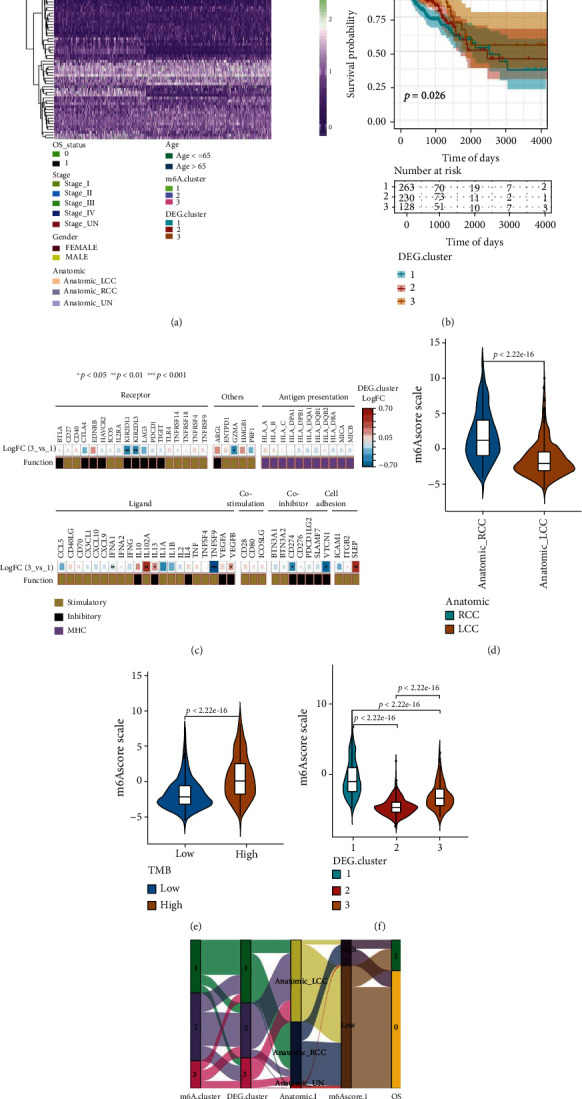
Identification and characterization of differential expressed gene (DEG) clusters. (a) A heat map of the expression profiles of DEGs. (b) Survival curves between DEG clusters. (c) Differential analysis of immunomodulatory molecule expression among DEG clusters. (d) A boxplot for m6Ascores across different anatomical locations. (e) A boxplot for m6Ascores between high- and low-tumor mutational burden (TMB) subgroups. (f) A boxplot for m6Ascores among DEG clusters. (g) A Sankey diagram illustrating the relationship of m6A cluster, DEG cluster, anatomical location, m6Ascore, and overall survival (OS).

Then, we sought to explore the correlation between the DEG clusters and clinical characteristics. As shown in Figure 6(a), the patients in Gene cluster-2 and Gene cluster-3 were younger than 65 years, had a better prognosis, and were diagnosed with left-sided colon and rectum cancer. (Figure 6(b)). Additionally, the differential expression of IMs among three clusters was analyzed, and the results indicated that multiple genes (including KIR2DL1, KIR2DL3, GZMA, IFNA2, IL12A, TNFSF9, VEGFA, VEGFB, CD274, VTCN1, and SELP) showed significant differential expression (Figure 6(c)). Then, we compared the m6Ascore in left- and right-sided samples and the results suggested much greater scores among the right-sided patients than those among the left-sided patients (Figure 6(d)). TMB is an important indicator to assess the efficacy of immunotherapy for CRC [13]. The results demonstrated a remarkably elevated m6Ascore among patients belonging to the high-TMB group compared to those belonging to the low-TMB group (Figure 6(e)). For m6A methylation DEG clusters, DEG cluster-1 demonstrated the highest median m6Ascore, while DEG cluster-2 demonstrated the lowest median m6Ascore (Figure 6(f)). These findings might provide novel insights into the mechanisms of m6A modifications and gene mutations among immune checkpoints in CRC. The association among m6A methylation regulators, DEG clusters, anatomical sites, m6Ascore, and OS is presented as a Sankey diagram (Figure 6(g)).

### 3.6. Validation of the m6Ascore Model in GEO Datasets

**Figure 7:**
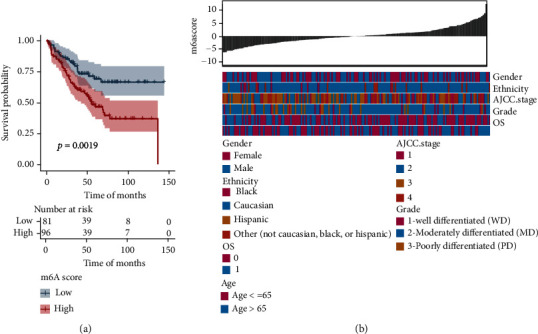
Evaluation of the predictive performance of m6Ascore in external datasets. (a) OS curves between high- and low-m6Ascore groups in the GSE17536 cohort. (b) A heat map depicting the correlation between m6Ascore and clinical characteristics in the GSE17536 cohort.

The robustness of the m6A risk model for predicting CRC prognosis was further validated on the GSE17536 dataset. The m6Ascore for the GSE17536 cohort was calculated using the previously screened DEGs. Next, the optimal density threshold of the m6Ascore associated with survival was calculated using the “survminer” R package, and the samples of GSE17536 were divided into high and low-m6Ascore groups. Results of the Kaplan-Meier analysis also revealed that the patients in the high-m6Ascore group had a poorer prognosis than those in the low-m6Ascore group (Figure 7(a), *P* < 0.01). Moreover, as shown in the heat map (Figure 7(b)), the m6Ascore could serve to assess several clinical features. The group with a high m6Ascore was associated with favorable histological subtypes and earlier staging in the GSE17536 cohort. All these findings showed that the m6Ascore model might be used to predict the prognosis and certain clinical parameters of patients with CRC and to provide potential therapeutic implications in the clinical setting.

### 3.7. Assessment of the Predictive Power of m6Ascore for ICI Immunotherapy Response

**Figure 8:**
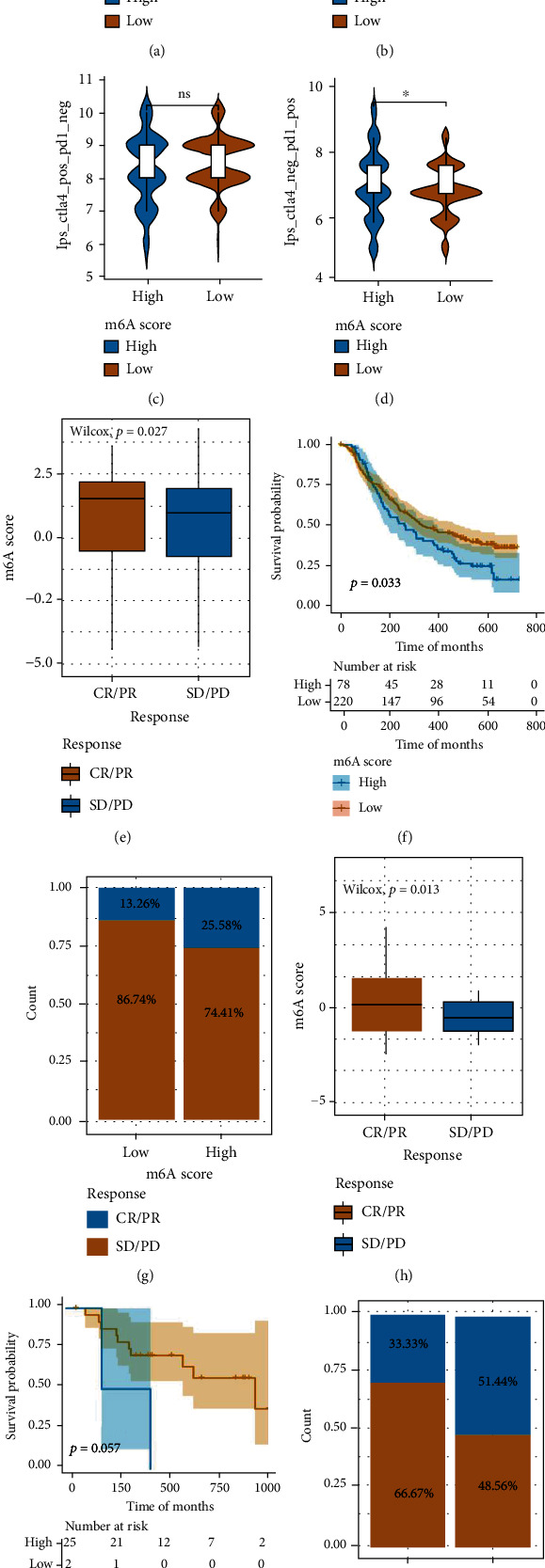
Evaluation of the performance of m6Ascore for predicting the clinical benefit of immunotherapy in patients with CRC. (a–d) Boxplot depicting differences in the four immunophenoscores between high- and low-m6Ascore subgroups. (e) Differences in m6Ascore among different clinical response subgroups in the IMvigor210 cohort. (f) OS curves between high- and low-m6Ascore subgroups in the IMvigor210 cohort. (g) Differences in clinical responses between high- and low-m6Ascore subgroups in the IMvigor210 cohort. (h) Differences in m6Ascore among different clinical response subgroups in the GSE78220 cohort. (i) OS curves between high- and low-m6Ascore subgroups in the GSE78220 cohort. (j) Differences in clinical responses between high- and low-m6Ascore subgroups in the GSE78220 cohort.

The immunophenogram was developed for predicting checkpoint blockade responses at a pancancer level by Charoentong et al. [39]. To determine the predictive power of the m6Ascore in terms of immunotherapeutic benefit, we obtained the dataset of TCGA-CRC cohort from The Cancer Immunome Atlas (TCIA) database and explored the correlation between immunophenoscore (IPS) and m6Ascore by performing immunophenogram analysis. The results illustrated that CTLA4-negative+PD-1-negative and CTLA4-positive+PD-1-positive cells exhibited significant differences in the immunotherapeutic response between the high- and low-m6Ascore groups (*P* < 0.05), whereas CTLA4-negative+PD-1-positive and CTLA4-positive+PD-1-negative cells exhibited no differences in the immunotherapeutic response between the high- and low-m6Ascore groups (*P* > 0.05). Moreover, the high-m6Ascore group tended to have a higher IPS score than the low-m6Ascore group, suggesting that patients in the high-m6Ascore group were more likely to gain benefit from immunotherapy (Figures 8(a)–8(d)).

Next, we investigated whether m6Ascore could predict the immunotherapeutic responses using the GSE78220 and IMvigor210 cohorts. Patients in the IMvigor210 cohort receiving anti-PD-L1 immunotherapy were classified into two groups according to the m6Ascore, namely, the high m6Ascore and the low m6Ascore. The findings demonstrated a significantly higher immunotherapeutic response rate in the high-m6Ascore group than that in the low-m6Ascore group (Figures 8(e) and 8(g)). Moreover, survival analysis demonstrated a significantly longer OS duration among patients in the low-m6Ascore group than that in the high-m6Ascore group (Figure 8(f)). Similarly, the same trend was found in the GSE78220 cohort, in which the patients were treated with anti-PD-1 immunotherapy (Figures 8(h)–8(j)). All these findings confirmed that m6Ascore might serve to predict the efficacy of immunotherapies and the prognosis of patients with CRCs.

## 4. Discussion

Recent studies have linked m6A modifications to the regulation of tumor immunity and response to ICIs [22]. To date, accumulating reports have shown the crucial roles of m6A regulators in carcinogenesis, development, diagnosis, therapy, and prognosis [21, 40]. However, the in-depth analysis of the m6A methylation regulator pattern and its significance on the TIME of CRC needs further elucidation. In this study, we shed light on the association between different m6A modification profiles and the immune landscape in CRC. This study improves our understanding of the TME and facilitates the development of more accurate immune therapy strategies for the treatment of patients with CRC.

In the present study, we first globally assessed somatic mutations, CNVs, and RNA expression of the 21 m6A regulators in TCGA-CRC cohort patients. We found that mutations and CNVs of the 21 m6A regulators were frequent in TCGA-CRC patients. At the transcriptomic level, we found that 2 m6A regulator expression was higher and 8 regulator expression was lower in TCGA-CRC patients. In addition, the correlation between each m6A regulators and TME-infiltrating cells was explored. Our results indicated that significant differences were observed between genes and immune cell infiltrations. Collectively, the results suggested that 21 genes encoding m6A regulators are highly heterogeneous in CRC patients and dysregulation of m6A regulators was associated with the development and antitumor immune response of CRC.

In addition, here, we focused on the regulatory role of the m6A reader YTHDC2 in colorectal cancer. To the best of our knowledge, the m6A readers have been investigated in other cancers [41–43], but YTHDC2 has been poorly studied in CRC. In the present study, the outcomes indicated that YTHDC2 was lowly expressed in colorectal cancer and coupled with prognostic analysis hinted that it might be a tumor suppressor, and this result was in line with Ji et al.'s results [44]. Further, YTHDC2 was significantly correlated with multiple immune cell infiltrations, and the expression of YTHDC2 gene in the high-TMB group was found to be significantly higher than that in the low-TMB group; hence, we inferred that it might be correlated with the efficacy of immunotherapy; unfortunately, it was not found after the validation using IMvigor210 dataset; the reason might be that this external dataset was not from CRC. Thus, in the future, CRC datasets need to be validated and basic experiments need to be performed to further investigate the mechanism of YTHDC2 in the regulation of immune cell infiltration.

Considering the highly individual heterogeneity of m6A regulator modification, then we uncovered the characteristics of m6A methylation modification mediated by 21 genes encoding m6A regulators in CRC. Based on 21 m6A regulators, we identified three distinct models of m6A modification by unsupervised consensus clustering in the CRC cohort. These three patterns termed m6Acluster-1, m6Acluster-2, and m6Acluster-3 are characterized by different prognoses and immune cell infiltrations in the TME. m6Acluster-1 presented a high proportion of effector TME immune cell activation and infiltration level, which are the hallmarks of the immune-inflamed phenotype, also described as a “hot” immune microenvironment [45]. Although m6Acluster-3 was also characterized by both elevated levels of TME immune cell infiltration, it had a high level of EMT1, EMT3, and pan-F-TBRS expression, which could determine the activation of stroma-associated pathways. Moreover, the patients exhibiting the m6Acluster-3 phenotype had worse survival compared to those exhibiting the m6Acluster-1 phenotype. The location and migration of T cells as well as the status of the stroma play crucial roles for antitumor immunosurveillance. In the case of high levels of immune cell infiltration, m6Acluster-3 could be classified as an immune rejection phenotype since T cells are restricted to the stroma, thus failing to penetrate the core tumor nest [46]. In contrast to m6Acluster-3 and m6Acluster-1, m6Acluster-2 was characterized by the lack of immune cell infiltration and immunosuppression, which could be labeled as an immune-desert phenotype. Previous research has suggested that this phenotype was probably correlated with the absence of activated and primed T cells and immune tolerance [47]. The immune rejection phenotype and immune-desert phenotype are also referred to as a “cold” immune microenvironment [48]. Charoentong et al. illustrated that the expression levels of IMs were closely associated with tumor genotypes and that tumor genotypes could determine tumor immunophenotypes and tumor escape mechanisms [39]. We found significant differences in the expression of IMs among the three m6A clusters. Collectively, the above findings confirmed the reliability of m6A modification patterns for identifying and classifying immune phenotypes of CRC.

Next, DEGs among the three m6A clusters were identified as m6A modification pattern-associated genes and were found to be enriched mainly in immune-related and stromal-related biological processes. Similar to the results of m6A patterns, three DEG clusters were recognized by unsupervised consensus clustering. These DEG clusters featured significantly different survival outcomes and IM expression. Moreover, we constructed a risk model, termed m6Ascore, for the purpose of quantifying the pattern of m6A modifications in patients with CRCs. Thus, the m6Ascore risk model could divide TCGA-CRC cohort into two groups, and survival analysis indicated that the patients belonging to the low-m6Ascore group had significantly longer OS duration than those belonging to the high-m6Ascore group. We employed the GSE17536 cohort to verify the reliability and robustness of the risk model in terms of OS prediction.

We next investigated whether m6Ascore is related to the TMB. The key initiator of adaptive immune activation is neoantigen recognition, and mutational profiles are suggested to be promising biomarkers to predict clinical response to ICI-based immunotherapy. However, it is difficult to detect the expression of overall neoantigens. TMB is easily available and commonly employed to estimate neoangiogenic load and is a good marker for predicting immunotherapeutic responses [49, 50]. The TMB was significantly higher among the patients in the high-m6Ascore group than those in the low-m6Ascore group. Subsequently, in both the IMvigor210 and GSE78220 cohorts, patients in the high-m6Ascore group demonstrated better responses to ICI immunotherapy in contrast with those in the low-m6Ascore group, which was consistent with the reports that patients with a high-TMB had a superior clinical response to immunotherapy. Furthermore, the m6Ascore can also be useful for the evaluation of clinical characteristics of patients, such as anatomical location, histological type, and tumor stages. Taken together, the above findings imply that m6Ascore is a reliable and robust biomarker for predicting the efficacy of ICI immunotherapies and is a promising tool to promote individualized immunotherapy for patients with CRC in the future.

There are a few limitations to the present study. First, the findings of this study are based on bioinformatic predictions; the m6A modification patterns and m6Ascore risk model were only established according to the 21 m6A regulator expression in TCGA-CRC cohort. Hence, further large-scale, multicenter, and prospective clinical cohorts are needed to verify our findings. Second, due to the lack of survival data of patients with CRC undergoing immunotherapy, the predictive reliability of m6Ascore in CRC needs further validation. Third, this study is a bioinformatics analysis and can be used as a preliminary reference. Further deep basic experimental researches are required to discover the association between m6A methylation and immune cell regulations, as well as the efficacy of immunotherapy in CRC cohort.

## 5. Conclusion

This study categorized CRC into three stable immune subtypes with distinct prognoses and TME based on 21 m6A methylation regulators. Importantly, a risk model-m6Ascore was developed to predict prognosis, clinical features, and the efficacy of immunotherapy in patients with CRC. The predictive power of the m6Ascore was validated on external datasets. Therefore, m6Ascore may be a viable tool to predict the immunotherapy response in patients with CRC, thus paving the way for personalized immunotherapy in the future.

## Data Availability

The data that support the findings of this study are available from the corresponding author upon reasonable request.

## Supplementary Materials

Supplementary Materials Figure S1: the workflow of our study design and analytical pipeline. Figure S2: consensus clustering of the expression profiles of 21 m6A methylation regulators in colorectal cancer (CRC). (A–D) Clustering results at classification numbers *k* = 2, 3, 4, and 5, respectively. (E) Distribution of CDF curves for consensus clustering. (F–I) Survival curves at classification numbers *k* = 2, 3, 4, and 5, respectively. (J) Distribution of area under cumulative distribution frequency (CDF) curves for consensus clustering. Figure S3: consensus clustering of differentially expressed genes (DEGs) among tumor m6A clusters. (A–D) Clustering results at classification numbers *k* = 2, 3, 4, and 5, respectively. (E) Distribution of cumulative distribution frequency (CDF) curves for consensus clustering. (F–I) Survival curves at classification numbers *k* = 2, 3, 4, and 5, respectively. (J) Distribution of area under CDF curves for consensus clustering.
