# Supplementary material for: Characterization of m6A Methylation Modification Patterns in Colorectal Cancer Determines Prognosis and Tumor Microenvironment Infiltration
Source: J Immunol Res. 2022 Jun 1;2022:8766735. doi: 10.1155/2022/8766735 (PMC9177296; doi:10.1155/2022/8766735)
Supplement: Supplementary Materials — Figure S1: the workflow of our study design and analytical pipeline. Figure S2: consensus clustering of the expression profiles of 21 m6A methylation regulators in colorectal cancer (CRC). (A–D) Clustering results at classification numbers k = 2, 3, 4, and 5, respectively. (E) Distribution of CDF curves for consensus clustering. (F–I) Survival curves at classification numbers k = 2, 3, 4, and 5, respectively. (J) Distribution of area under cumulative distribution frequency (CDF) curves for consensus clustering. Figure S3: consensus clustering of differentially expressed genes (DEGs) among tumor m6A clusters. (A–D) Clustering results at classification numbers k = 2, 3, 4, and 5, respectively. (E) Distribution of cumulative distribution frequency (CDF) curves for consensus clustering. (F–I) Survival curves at classification numbers k = 2, 3, 4, and 5, respectively. (J) Distribution of area under CDF curves for consensus clustering. [file 8766735.f1.zip › Supplementary Figure legends.docx]

**Supplementary Figure legends：**

Figure S1: The workflow of our study design and analytical pipeline.

Figure S2. Consensus clustering of the expression profiles of 21 m6A methylation regulators in colorectal cancer (CRC). (A-D) Clustering results at classification numbers k=2, 3, 4, and 5, respectively. (E) Distribution of CDF curves for consensus clustering. (F-I) Survival curves at classification numbers k=2, 3, 4, and 5, respectively. (J) Distribution of area under cumulative distribution frequency (CDF) curves for consensus clustering.

Figure S3. Consensus clustering of differentially expressed genes (DEGs) among tumor m6A clusters. (A-D) Clustering results at classification numbers k=2, 3, 4, and 5, respectively. (E) Distribution of cumulative distribution frequency (CDF) curves for consensus clustering. (F-I) Survival curves at classification numbers k=2, 3, 4, and 5, respectively. (J) Distribution of area under CDF curves for consensus clustering.
