# Supplementary material for: Characterization of m6A Methylation Modification Patterns in Colorectal Cancer Determines Prognosis and Tumor Microenvironment Infiltration
Source: J Immunol Res. 2022 Jun 1;2022:8766735. doi: 10.1155/2022/8766735 (PMC9177296; doi:10.1155/2022/8766735)
Supplement: Supplementary Materials — Figure S1: the workflow of our study design and analytical pipeline. Figure S2: consensus clustering of the expression profiles of 21 m6A methylation regulators in colorectal cancer (CRC). (A–D) Clustering results at classification numbers k = 2, 3, 4, and 5, respectively. (E) Distribution of CDF curves for consensus clustering. (F–I) Survival curves at classification numbers k = 2, 3, 4, and 5, respectively. (J) Distribution of area under cumulative distribution frequency (CDF) curves for consensus clustering. Figure S3: consensus clustering of differentially expressed genes (DEGs) among tumor m6A clusters. (A–D) Clustering results at classification numbers k = 2, 3, 4, and 5, respectively. (E) Distribution of cumulative distribution frequency (CDF) curves for consensus clustering. (F–I) Survival curves at classification numbers k = 2, 3, 4, and 5, respectively. (J) Distribution of area under CDF curves for consensus clustering. [file 8766735.f1.zip › Supplementary Table S1.pdf]

Supplementary Table S1

Table S1. The 21 N^6-methyladenosine (m^6A) regulators

Gene symbol	Full Name	Category
METTL3	Methyltransferase-like protein 3	Writer
METTL14	Methyltransferase-like protein 14	Writer
METTL16	Methyltransferase-like protein 16	Writer
WTAP	Wilms tumor 1-associated protein	Writer
VIRMA (KIAA1429)	Vir-like m^6A methyltransferase associated protein	Writer
ZC3H13	zinc finger CCCH domain-containing protein 13	Writer
RBM15	RNA-binding motif protein 15	Writer
RBM15B	RNA binding motif protein 15B	Writer
YTHDC1	YTH domain-containing 1	Reader
YTHDC2	YTH domain-containing 2	Reader
YTHDF1	YTH m^6A RNA-binding protein 1	Reader
YTHDF2	YTH m^6A RNA-binding protein 2	Reader
YTHDF3	YTH m^6A RNA-binding protein 3	Reader
HNRNPC	Heterogeneous nuclear ribonucleoprotein C	Reader
HNRNPA2B1	Heterogeneous nuclear ribonucleoprotein A2B1	Reader
IGF2BP1	Insulin-like growth factor 2 binding protein 1	Reader
IGF2BP2	Insulin-like growth factor 2 binding protein 2	Reader
IGF2BP3	Insulin-like growth factor 2 binding protein 3	Reader
RBMX	X-linked RNA-binding motif protein	Reader
FTO	Fat mass and obesity-associated protein	Eraser
ALKBH5	Alk B homologue 5	Eraser
